# Plant–Fungi Interactions: Where It Goes?

**DOI:** 10.3390/biology12060809

**Published:** 2023-06-02

**Authors:** A. K. Hasith Priyashantha, Dong-Qin Dai, Darbhe J. Bhat, Steven L. Stephenson, Itthayakorn Promputtha, Prashant Kaushik, Saowaluck Tibpromma, Samantha C. Karunarathna

**Affiliations:** 1 Center for Yunnan Plateau Biological Resources Protection and Utilization, College of Biological Resource and Food Engineering, Qujing Normal University, Qujing 655011, China; priyashanthahasith@gmail.com (A.K.H.P.); cicidaidongqin@gmail.com (D.-Q.D.); 2 Department of Botany and Microbiology, College of Science, King Saud University, P.O. Box 2455, Riyadh 11451, Saudi Arabia; bhatdj@gmail.com; 3 Biology Division, Vishnugupta Vishwavidyapeetam, Gokarna 581326, India; 4 Department of Biological Sciences, University of Arkansas, Fayetteville, AR 72701, USA; slsteph@uark.edu; 5 Department of Biology, Faculty of Science, Chiang Mai University, Chiang Mai 50200, Thailand; itthayakorn.p@cmu.ac.th; 6 Independent Researcher, 46022 Valencia, Spain; prakau@alumni.upv.es; 7 National Institute of Fundamental Studies (NIFS), Hantana Road, Kandy 20000, Sri Lanka

**Keywords:** ascomycota, phytopathogenic fungi, plant defense mechanism, symbiotic fungi

## Abstract

**Simple Summary:**

Fungi can form several different types of associations such as pathogenic and symbiotic with plants. Pathogenic fungi subject plants to a great deal of pressure by causing various diseases. Unlike animals, plants cannot escape from biotic and abiotic threats by moving from one place to another, but they survive such alarming conditions in some wonderful ways. In turn, plants enjoy the interactions with symbiotic fungi as they offer many benefits to the plants. Changes in micro- and macro-climates lead to modifying the interactions between plants and fungi, either positively or negatively. In this paper, we discuss all of those interactions and their relevance to better agricultural practices.

**Abstract:**

Fungi live different lifestyles—including pathogenic and symbiotic—by interacting with living plants. Recently, there has been a substantial increase in the study of phytopathogenic fungi and their interactions with plants. Symbiotic relationships with plants appear to be lagging behind, although progressive. Phytopathogenic fungi cause diseases in plants and put pressure on survival. Plants fight back against such pathogens through complicated self-defense mechanisms. However, phytopathogenic fungi develop virulent responses to overcome plant defense reactions, thus continuing their deteriorative impacts. Symbiotic relationships positively influence both plants and fungi. More interestingly, they also help plants protect themselves from pathogens. In light of the nonstop discovery of novel fungi and their strains, it is imperative to pay more attention to plant–fungi interactions. Both plants and fungi are responsive to environmental changes, therefore construction of their interaction effects has emerged as a new field of study. In this review, we first attempt to highlight the evolutionary aspect of plant–fungi interactions, then the mechanism of plants to avoid the negative impact of pathogenic fungi, and fungal strategies to overcome the plant defensive responses once they have been invaded, and finally the changes of such interactions under the different environmental conditions.

## 1. Introduction

Multiple and intricate interactions between plants and fungi do exist in nature [1,2,3]. Plants are always prone to interact with various microbes in different ways which include phytopathogenic and symbiotic associations. In phytopathogenic associations fungi interact with different lifestyles, namely necrotrophic (e.g., *Alternaria alternata*, *A. solani*, *A. brassicae*, *Aspergillus flavus*, *Bipolaris sorokiniana*, *Botrytis cinerea*, *Claviceps gigantean*, *Colletotrichum beeveri*, *C. gloeosporioides*, *C. graminicola*, *C. musae*, *Sclerotinia sclerotiorum*, *Stenocarpella maydis*, *Zymoseptoria tritici*) [4,5,6,7,8,9,10,11,12,13,14,15,16]; biotrophic (e.g., *Blumeria graminis*, *Cladosporium fulvum*, *Hemileia vastatrix*, *Melampsora lini*, *Phakopsora pachyrhizi*, *Puccinia arachidis*, *Puccinia graminis*, *Puccinia kuehnii*, *Puccinia striiformis*, *Sporisorium scitamineum*, *Ustilago maydis*) [17,18,19,20,21,22,23,24,25,26,27,28]; and hemibiotrophic (e.g., *Colletotrichum higginsianum*, *C. trifolii*, *Fusarium equiseti*, *F. oxysporum*, *F. sacchari*, *Ganoderma boninense*, *Magnaporthe oryzae*, *Phomopsis longicolla*) [29,30,31,32,33,34,35,36]. A plethora of fungi also live as symbiotic, e.g., *Funneliformis mosseae*, *Glomus albidum*, *G. etunicatum*, *G. mosseae*, *G. fasciculatum*, *Glomus albidum*, *G. etunicatum*, *G. mosseae*, *G. fasciculatum*, *Glomus mosseae*, *Trichoderma virens* [37,38,39,40,41,42,43].

These interactions may significantly impact agriculture, the environment and, ultimately, the economy [44]. Interactions between plants and fungi can be highly effective in shaping plant community composition and diversity because they can affect the plants directly as well as indirectly by affecting competition and facilitation [45].

### 1.1. Overview of Phytopathogenic Fungi

The majority of fungal phytopathogens belong to the phyla Ascomycota and Basidiomycota. Plant pathogens are classified in a number of classes among Ascomycetes, such as the Dothideomycetes (including *Cladosporium* spp.), Sordariomycetes (including *Magnaporthe* spp.), or the Leotiomycetes (e.g., *Botrytis* spp.). Rusts (Pucciniomycetes) and smuts (spread among the subphylum of Ustilaginomycotina), the two major plant pathogen groups, are members of the Basidiomycetes [46].

Pathogenic fungi interact negatively with plants, can infect all parts of the plant and even kill them, and are responsible for causing ecologically and commercially significant plant diseases [5,6]. One of the most striking examples is *Botrytis cinerea*, which can infect and cause disease in more than 1000 species of plants, including many fruits, flowers, and leafy vegetable crops [10]. It has also been reported that the economic losses due to *B. cinerea* exceed USD 10 billion worldwide annually [9]. Further, in Sichuan Province, China disease caused by pathogens (e.g., *Blumeria graminis*) has resulted in wheat yield losses of 5 to 8% in general, and in an even more severe stage it goes up to 100% [19]. As aforementioned, plant pathogens are frequently grouped according to their lifestyle with their host plants. Necrotrophs infect living plants and destroy infected tissues as soon after they invade. The necrotrophs obtain nutrition from hosts, leading to the death of the affected organ or the entire plant [47]. Biotrophs infect living plants by weakening the plant’s immune system and obtaining nutrients from the living cells. Hemibiotrophs first establish infection in living plant tissues, similar to biotrophs, and then uptake the nutrients from a combination of feeding from living and killed host cells [47,48]. Plants defend against those devastating pathogens through various mechanisms. Passive defense mechanisms are radially available against the pathogens, even before they contact the host. In these cases, physical barriers (e.g., cuticle, cell wall, stomatal aperture, and lenticels) and chemical barriers (e.g., pH, nutrient deprivation, and phytoanticipins) commonly work against the phytopathogenic fungi. Another type, the active defense mechanism (which we discuss in this article), is activated only after pathogen recognition and is mainly classified into rapid active defense reactions and delayed active defense reactions. Rapid active defense responses include the changes in membrane permeability (e.g., generation of reactive oxygen species or ROS, hydrogen peroxide or H_2_O_2_); hypersensitive cell death (HR); a fast, localized kind of programmed cell death; and cell wall fortification (e.g., papilla/callose deposition). Delayed active defense responses include wound repair (e.g., layers of cork cell formation), expression of pathogenesis-related (PR) proteins (e.g., accumulation of chitinase), and systemic acquired resistance (e.g., salicylic acid (SA) biosynthesis) [49].

Compatible interactions occur when a plant fails to coordinate effective defense responses, enabling the disease to establish and spread throughout the host. On the other hand, an incompatible interaction occurs when the host’s active defense mechanisms effectively stop the spread of the pathogen within the tissues of the host [50]. However, pathogens do not always create an interaction with the host. In fact, germination of a fungal spore can occur even in the absence of a host. The hypha may not penetrate the host tissues due to the passive defense mechanism of the host and ultimately die off. Thus, the fungus fails to establish a pathogenic relationship [51,52].

### 1.2. Overview of Symbiotic Fungi 

In general, symbiosis refers to any type of close and long-term interaction between different organisms. Fungi live symbiotically, in general. They may interact with the plant, by either commensalism (one organism benefits from it and the other one shows no apparent beneficial or harmful effect) or mutualism (both gain reciprocal benefits and are usually mutual). In addition, some symbiotic fungi also can create harmful effects on the plant under certain conditions at some stage of their lifecycle, thus they are called pathogenic [3].

Among the symbionts, mycorrhizal glomalean fungi are one of the most studied groups. They can be divided into several different relationship types at the coarsest level, including arbuscular mycorrhizal (AM), ectomycorrhizal (EcM), and ericoid mycorrhizal (ErM) [53]. Amongst them, AM fungi, belong to Glomeromycota and are the most prevalent endomycorrhizal (e.g., intracellular) mutualists with most vascular plants. They are also the earliest endomycorrhizal fungi to date and are found to be associated with about 85% of living plant species [54]. At the same time, EcM fungi are a huge group with a wide distribution, although they are only connected with 3–4% of the vascular plant families. Chiefly, they are members of the phyla Ascomycota and Basidiomycota [55]. However, ErM fungi are associated with a restricted diversity of plant species in the Ericaceae, Epacridaceae, and Empetraceae. In addition, they belong to a restricted group of fungi of the Ascomycetes [56]. In contrast to phytopathogens, symbiont interaction gives significant benefits to plants. Symbiont fungi enhance the structure and aggregation of the soil, which in turn influences the organization of plant communities and production.

Symbiotic fungi colonize the rhizosphere region in soil or the plant’s internal tissues, usually take carbon (C) from the host plant and return to the plants with essential soil elements, and also improve the water and nutrition uptake by the plant [57,58]. Especially, some ErM and EcM secrete protease and phosphatases that access organic nitrogen (N) and phosphorus (P) to plants. In addition, some of them can also produce plant cell wall degrading enzymes, facilitating access to more organic N and P [59]. Moreover, they improve the N fixation, P solubilization, sulfur (S) oxidization, plant hormone production, or decomposition of organic compounds and further act as biofertilizers (e.g., *Alternaria* spp., *Aspergillus* spp., *Chaetomium* spp., *Fusarium* spp., *Penicillium* spp., *Serendipita* spp., *Phoma* spp, and *Trichoderma* spp.) [2,58]. In addition to those, symbiosis helps plants to retaliate against the biotic (e.g., parasites) and abiotic (e.g., drought, salinity, toxic compounds, and flooding) [54,57] stresses. Noteworthy that in addition to those symbionts, endophytic fungi also offer the aforementioned same benefits, while entomopathogenic fungi protect plants from insect pest attacks and also stimulate the plant defense responses [60,61,62]. However, unfortunately, in some cases, symbionts can also increase the activity of other plant pathogens, such as viruses (e.g., Potato virus), thus increasing disease susceptibility and reducing plant height and root development [41]. 

Accounting all, it is clear that their significance particularly in the agriculture sector, is not only because of their influence on improving farm productivity [63], but also because of their ability to minimize the application of agrochemicals. This leads to multi-dimensional effects, including the degradation of the environment, health complications, and development of pest-resistance [64,65], therefore direct to sustainable agro farming [66,67,68].

In this article, we attempted to give an overall picture of the plant–fungi interaction, particularly addressing the phytopathogenic and symbiotic fungi. In order to acquire the relevant information, an extensive literature survey was also conducted by referring to the most recent studies wherever possible. Thus, the paper is worthy of up-to-date information. First, we provided the background data (other than in the introduction) that support the main content of this article, especially by sharing the evolutionary aspect of plant–fungi interactions. Second, we elaborated on how plants defend against pathogenic fungi and how fungi try to overcome those defense responses to create beneficial interactions. 

According to the literature, the alteration of plant–fungi interaction under the changing environmental condition appears to be an emerging work of study. In addition, not enough comprehensive analyses have been completed in this regard. Therefore, this paper, most importantly, discusses how the broad group of fungi may change their interaction with plants due to the variation of environmental conditions, also bringing the most recent advancement in the field.

## 2. The Evolutionary Aspect of Plant-Fungal Interaction

### 2.1. Phytopathogenic Fungi

It is thought that fungal–plant association evolved at least 450 or 460 million years ago, most probably with the symbiotic fungi [69,70]. Unlike symbiotic fungi, diversion of phytopathogenic fungi occurred (relatively) recently [71]. It is observed that phytopathogenic fungi are unevenly distributed (phylogenetically) throughout the fungal kingdom [72]. The antagonistic interaction between plants and their diseases produces co-evolutionary dynamics in which plants respond to recognize the pathogens, and pathogens evolve to escape plant defense mechanisms in natural environments. Phytopathogenic fungi show high rates of molecular evolution, and their capacity to cope and adapt to the new environment ensures their survival throughout history [73]. Van Valen [74] presented the ‘Red Queen hypothesis’ (RQH) emphasizing the primacy of biotic interactions over abiotic forces in driving evolution. According to the RQH, any adaptation made by one species is countered by adaptations made by another interacting species, so constant evolutionary change is required for survival. Thereafter, several additional explanations were made by the scientists to the RQH. Today, collective theories provide a more conceptual framework for the evolution of a suite of characteristics such as mating systems, pathogen virulence, host resistance, and the maintenance of population genetic diversity [75,76,77]. For instance, in mating systems, the majority of phytopathogenic fungi can reproduce sexually or asexually, exclusive sexual and asexual species are significantly minor. In the case of asexuality, it has been hypothesized to arise frequently from sexual fungal species, during the evolution time frame [78]. In the sexual reproduction system, one of the most important benefits is that it generates genetic variation among the progenies, which may allow the population to adapt faster to novel and/or stressful environments through rapid adaptation to genetic variation. In spite of this, adaptation to stressful environments or new environments can still be accomplished without the involvement of sex through mutations [79]. Moreover, beneficial natural mutations can be detached from the deleterious mutations in sexual populations and can be evolved as separate lineages, thus taking evolutionary advantage [80]. A recent study conducted by Meng et al. [4] indicated that *Alternaria alternata*, combining many cycles of asexual propagation with fewer cycles of sexual reproduction, thereby enabling it to adapt to changing environments. In turn, species such as *Zymoseptoria tritici* which have limited asexuality showcase a number of mutations at a single nucleotide position leading to a higher level of genetic diversity. Moreover, new phenotypes evolve in a highly selective environment (e.g., monoculture of host plant) where the trait is desirable. In that case, the proportions of new phenotypes in the population can rapidly increase [18]. 

### 2.2. Symbiotic Fungi

Symbiotic interaction between plant fungi is unavoidable as same as connecting with pathogens. It is also worth noting that, unlike mycorrhizae, most fungal endophytes are considered commensalistic and can have either positive (mutualists) or rarely negative (pathogens) interactions with host plants. According to fossil records and recent genetic analysis, it is clear that in both mycorrhizal and endophytes symbiosis likely played a role in the early colonization of the land by plants, serving as the evolutionary cornerstone of the current flora of land plants [54,59]. Therefore, the historical bond of symbiotic interaction of plants with mycorrhizal and endophytic fungi runs over 460 [69] and 400 million [81] years back, respectively.

In an evolutionary scenario for mycorrhizal symbiosis, members of Zygomycota had a significant role in the early phases of land plant diversification. Later on, Mucoromycotina was replaced with Glomeromycotina in most plant lineages. It is believed that Glomeromycotina has transitioned into various Ascomycota and Basidiomycota lineages, leading to the emergence of several new mycorrhizal syndromes, e.g., orchids [82]. More precisely, among the mycorrhizal groups, AM and EcM fungi show clear differences in their evolutionary origins. AM symbiosis in plants and fungi has a single origin, with subsequent losses and sporadic reversions back to AM in the seed plants. EcM symbiosis shows multiple, independent evolutionary origins in both plants and fungi [83]. Furthermore, accounting for the AM, owing to their relatively simple spores, lack of sexual reproduction, and association with a wide range of plants, they are considered primitive. In addition, they are mostly unable to colonize without plants [84]. According to fossil records, the origin of glomalean fungi runs 55 to 60 million years ago. Surprisingly, those evolved before the vascular plants arose and association with non-vascular plants such as Bryophyta (e.g., Hornworts) was found in early ages. However, they might have evolved into saprobes or developed Geosiphon (a non-mycorrhizal ancestral member of the Glomales, that formed an endosymbiosis with cyanobacteria)-type symbioses [69]. This ancient AM relationship is still present in 80% of plant species, proving the value of this mutualism to both parties [85]. In brief, endophytic fungi have been divided into two major groups namely, clavicipitaceous and non-clavicipitaceous endophytes. Clavicipitaceous endophytes infect some grasses limited to cool regions, while non-clavicipitaceous are found to be associated with non-vascular plants, ferns and allies, conifers, and angiosperms. Endophytes are also restricted to the division of Ascomycota and Basidiomycota [86].

It is also important to know what the possible reasons for such an association could be. It is accepted that early plants having poor development of roots or lack of true roots make it difficult to absorb the necessary nutrition, thus putting pressure on their survival. Therefore, it is evident that the evolution of plants with symbiosis was primarily aimed at large-scale colonization of the land [59]. In turn, EcM fungi have evolved from saprophytic fungi. This understanding is supported due to their production of enzymes that have the potential to digest plant cell walls, though these generally occur at considerably lower levels than in saprophytic fungi [84]. It has also been recognized that these symbionts have evolved to actively suppress the host’s defense response [87]. 

## 3. Plant–Fungal Interactions: Heaven or Hell 

### 3.1. Plant Defense Mechanism in Plant–Fungi Interaction

In response to the pathogen attack, plants impose defense mechanisms to protect themselves. Switching to such a defense mechanism also has negative consequences for the plants as it can negatively influence plants’ growth. Nonetheless, plants may survive as it is the primary need of such a mechanism. Gene-for-gene (GFG) identification of the pathogen frequently marks the start of the responses towards immunological responses, which are triggered by the identification of pathogen-associated molecular patterns (PAMPs) or microbe-associated molecular patterns (MAMPs), by plant pattern recognition receptors (PRRs) localized in the plasma membrane and are mainly found in the forms of receptor-like protein kinases and receptor-like proteins [88,89,90]. MAMPs molecules are crucial for the fitness and survival of microbes and are conserved across species, giving plants an effective way to detect the contact of pathogens. In the case of fungal contact, plants secrete chitin-producing enzymes called chitinases to release chitin fragments (chitin oligomers) from the cell wall. Then, it serves as an elicitor in MAMP [91]. For example, Hawkins et al. [92] identified seven chitinase genes in maize (*Zea mays*) that had alleles associated with increased resistance against the *Aspergillus flavus* infection. PRRs trigger the immune response and are called PAMP-triggered immunity (PTI), which provides a shield against non-host pathogens and minimizes the disease caused by virulent pathogens [89]. Further addressing, activation of PRRs signaling causes accumulation of reactive oxygen intermediates; activation of ion channels; activation of specific, defense-related mitogen-activated protein kinase cascades; and extensive transcriptional reprogramming of the host [93]—which altogether leads to accumulation of antifungal compounds such as phenols and phenolic glycosides, unsaturated lactones, sulfur compounds, saponins, cyanogenic glycosides, and glucosinolates [94]. R proteins have two conserved features, nucleotide-binding (NB) and leucine-rich repeat (LRR) domains, collectively referred to as NLRs. A wide range of NLRs is used by host plants to rapidly detect fungal effectors, which suppress the plant immunity responses’ interaction with host proteins to modulate plant metabolism, either producing harmful secondary metabolites or proteins that kill the host plant during pathogen invasion. NLRs either directly or indirectly recognize the effectors, and this recognition frequently triggers an HR [90,93,95]. Moreover, HR often results through the activation of complex signal transduction pathways, case in point, ERK-like (extracellular-signal-regulated kinase) mitogen-activated protein kinases (MAPKs) [14]. It is widely recognized that the immune responses elicited by PRRs and NLRs are similar. Nevertheless, differences exist in the duration and amplitude of ETI responses, which are generally considered more significant than those of PTI (PAMP-triggered immunity) [90]. PTI and ETI extensively overlap, also signaling likely interactions [96]. 

The activation of plant defensive responses mediated by molecular mechanisms is enormously complex. In species, numerous biotrophic pathogens interact with their hosts in a framework of GFG that is traditionally described in which plant disease resistance (*R*) genes recognize the products of specific avirulent (*Avr*) genes in pathogens, resulting in disease resistance. For example, the first fungal avirulence gene—*Avr9* in *Cladosporium fulvum*—causes disease in tomatoes [97]. In these interactions, the disease is caused by a lack of either the *R* gene or the matching *Avr* gene [98,99]. An oxidative burst, or the rapid generation of ROS, typically occurs in conjunction with *R* gene-mediated resistance. Other than the host-induced ROS, some pathogens (e.g., *Alternaria brassicae*) produce those ROS during the compatible interaction at the hyphal tips [6]. Note that this production of ROS is also needed for HR response. The expression of several pathogenesis-related (PR) proteins, which are hypothesized to contribute to resistance, is linked to the activation of a signaling pathway dependent on salicylic acid (SA) in *R* gene-mediated resistance [100]. Other plant defensive responses are regulated by jasmonates (JAs), salicylic acid (Sa)-and/or ethylene-dependent systems (ET) [101]. It also needs to be highlighted that JAs represent the jasmonic acid (JA) and its derivatives, including its methyl ester (MeJA) and amino acid isoleucine conjugate (JA-Ile). JA improves plant disease tolerance through the JA signaling pathways in general, and aforementioned PAMPs are also associated with the JA signaling pathways [102]. JA and ET are typically linked to the defense against necrotrophic development. In contrast, the activation of defense mechanisms against biotrophic and hemibiotrophic diseases and the development of systemic acquired resistance are all facilitated by SA, which plays a crucial role in the plant defense system. Despite the fact that the SA and JA/ET defense pathways are antagonistic to one another, evidence of synergistic interactions has been found [101]. In a study, Tamaoki, et al., [103] attempted to understand the activation of the common defense mechanism of rice via JA and SA. According to them, SA signaling mainly contributes to the basal defense system in normal conditions due to the high endogenous SA concentrations. Nevertheless, sharp drops of endogenous SA are reported once the JA signal is switched on, and SA signaling is found to be suppressed. Then, instead of SA, JA turns on the common defensive system. In contrast, Pena-Cortés et al. [104] found that SA blocks the biosynthesis of JA in tomato leaves. Thus, it is clear that the production of SA and JA is antagonistically inhibited. In a comprehensive study, Riemann et al. [105] show the involvement of JA derivative in rice defense response against the blast fungus, *Magnaporthe oryzae*. They have highlighted the role of exogenous JA, inducing the production of phytoalexins, which are important antimicrobial secondary metabolites that are produced in response to pathogen infection. Further, Riemann et al. [105] found that the JA-independent pathway worked against the *M. oryzae* induced accumulation of phytocassanes and partially for the accumulation of momilactones. Thus, it is conceivable that phytoalexin buildup is mediated by JA in the defense against blast fungal infection.

### 3.2. Fungi Overcome Plant Defense Mechanism in Plant–Fungi Interactions

Recent studies have shown that interactions between necrotrophs and their host plants are much more nuanced and intricate than previously thought [106]. For instance, when compared to biotrophs, some necrotrophs express effector proteins that are internalized by host cells and interact with the host in a GFG manner to cause disease, by suppressing or avoiding host basal defense or PAMP-triggered immunity (PTI) [107].

Conversely, the initial pathogenesis phase does not differ among the hemibiotrophic and biotrophic fungi (obligate), though various mechanisms are applied to take up the nutrients from the host. It is well understood that compared to the biotrophic and hemibiotrophic fungi, necrotrophs have broader host ranges and overcome the natural physical barriers of the plants (e.g., cell wall) through the secretion of toxins and extracellular cell wall degrading enzymes, which degrade a wide range of complex and cross-linked polysaccharides and glycoproteins [108]. 

In particular, *Alternaria alternata* produces the most prominent determinant of their virulence. The host-selective toxin (HST) called ACT (9,10-epoxy-8-hydroxy-9-methyl-decatrienoic acid) induces rapid electrolyte leakage from citrus cells. Furthermore, the release of cutinase, cellulose, and pectate lyase enzymes degrade the cells and form necrotic lesions [109]. Unlike necrotrophs, biotrophic fungi predominantly take up the necessary nutrients by creating invading structures of melanized appressoria, penetration hyphae, and infection hyphae to make a closer association with the host and to also avoid extreme damage within active plant cells [110]. In order to enter into leaf epidermal cells, some fungi (e.g., *Magnaporthe oryzae*) generate tremendous turgor pressure in the appressorium, and this pressure is used to puncture the host surface with a thin penetration peg, leading to the further development of the fungi inside the tissue [35].

As aforementioned, hemibiotrophs follow a short biotrophic phase and absorb the nutrients similarly once keeping the host cells alive. Next, they switch into the destructive necrotrophic development characterized by extended secondary hyphae that grow both intracellularly and intercellularly and cause the death of tissues [111,112]. In some cases, hemibiotrophs such as *Colletotrichum lindemuthianum* mutant H433 retain biotrophically and will not be moved to the necrotrophic phase [111]. Nonetheless, the difference between the necrotrophs and biotrophs during the initial infection process is quite interesting. As aforesaid, cell death is obvious at the primary stage of the pathogenic infection in both pathogenic types; however, cell death has remarkably different roles in plant responses to necrotrophs and biotrophs. While cell death caused by the necrotrophs is a sign of successful infection, in biotrophs, HR-associated cell death or the death of cells at the place of infection is actually a plant defense response where it stopped the further spread of pathogen hyphae, thereby limiting the nutrient intake to the pathogen which restricts their further development. In other words, necrotrophs aggressively encourage cell death by utilizing a variety of virulence factors, whereas biotrophs actively inhibit HR cell death [113]. Intriguingly, HR is also associated with the hemibiotrophs such as *Phytophthora infestans*, even though they show necrotrophic action in a later stage [114].

In the interest of endophytic fungi, many studies conducted to evaluate how they counteract the defense response of plants leading to inter and intracellular colonization. Endophytes are initially perceived as potential invaders. Like pathogens, they also have evolved sophisticated strategies to avoid recognition and elude plant immune systems. More precisely, endophytic fungi need to avoid either eliciting PTI or adapting to it or suppressing it to establish a compatible interaction that leads to proliferation [61,87,93]. For example, as previously highlighted, in plants, chitinases are effective immune molecules, as they break down chitin and weaken fungal cell walls. This type of detection by the plant immune system has to be prevented by the fungi, and they do so in two ways: (1) fungi could mask the chitin in their cell wall by covering it with other polymers or deacetylating it into chitosan and (2) they modify the elicitor-active chitin oligomers produced by the chitinases involved in the plant’s immune response. These chitin oligomers may be inactivated by being bound, degraded, or deacetylated. In both cases, deacetylation appears to be the most likely inactivator of chitin since it is known that fully deacetylated chitosan oligomers do not bind to plant receptors and therefore do not induce an immune response [1]. On the other hand, fungi also must either inactivate toxic metabolites or secrete effectors to accomplish further survival in or inside the plant host. The effectors can also release proteins that act as a barrier for the fungus, reducing the host’s immunological response, or altering the physiology of the host cell [93]. Some pathogens—for instance, *Sclerotinia sclerotiorum*—kill host plants via secretion of small, toxin, effector-like protein identified as SsSSVP1 and are expected to form disulfide bonds intra-molecularly [115]. As those effectors are proteins, they also export in fungi encompassing the signal peptide-mediated transfer through the endomembrane system. They first enter into the endoplasmic reticulum (ER), undergo proper folding, translocate the effectors to the Golgi apparatus, pack into secretory vesicles, fuse with the cytoplasmic membrane, and finally release into the extracellular space. This conventional endoplasmic reticulum–Golgi apparatus route is the most opted pathway to secrete [116]. In general, to secrete the effectors, contact should be there between the fungi and plants. Haustoria and hyphae are both secretion effectors. Primarily, localized release of those effectors can be observed at the interface between fungal pathogen and host plant, also associated with the penetration pore. This is important for hemibiotrophs since this stage leads to biotrophic changes to a necrotrophic lifestyle [29]. Those effectors then pass through the extra-haustorial matrix, extra-haustorial membrane for effectors secreted from haustoria, and apoplast, plant cell wall, and plant plasma membrane for effectors secreted from hyphae, then translocated into the host cell cytoplasm, although the followed mechanisms are still unclear [117].

One of the widespread effectors called necrosis-and ethylene-inducing-like proteins (NLPs) contributes to pathogen virulence through phytotoxic activity [118]. In this regard, Santhanam and co-workers [119] demonstrated the cytotoxic activity of NLP family members of a tomato-pathogenic *Verticillium dahliae* strain and found that two of the seven NLP-induced plant cell death. In *V. dahliae*, the genes encoding these cytotoxic NLP are found to be induced upon colonization of tomato. In addition to their role in virulence, Santhanam et al. [119] have found that one of the NLP genes also contributes to vegetative growth and conidiospore generation (asexual reproduction). In a similar study, Kombrink et al. [120] studied Chitin-binding lysin motif (LysM) effectors that contribute to the virulence of *V. dahliae*, which are causal agents of foliar diseases on various plants including Arabidopsis, tomato, and *Nicotiana benthamiana*. Most intriguingly, Kombrink and co-workers [120] found that the LysM effector binds chitin, has the ability to block immunological responses, and shields fungal hyphae from degradation by hydrolytic plant enzymes.

Another important consideration is that the fungi can also overcome the defense responses by inactivating previously mentioned endogenous JA through the biosynthesis of monooxygenase (Abm) to hydroxylate it, where such findings revealed studies along with *Magnaporthe oryzae*. More closely, Abm converts endogenous free JA into 12OH-JA. This 12OH-JA is released during pathogen-host penetration and helps to avoid a defense response. Importantly, after the invasion, Abm itself is secreted and likely changes plant JA into 12OH-JA to aid host colonization [121].

## 4. Plant–Fungal Interactions under the Changing Environmental Conditions

### 4.1. Environmental Factors and Plant–Fungi Interaction

**Figure 1:**
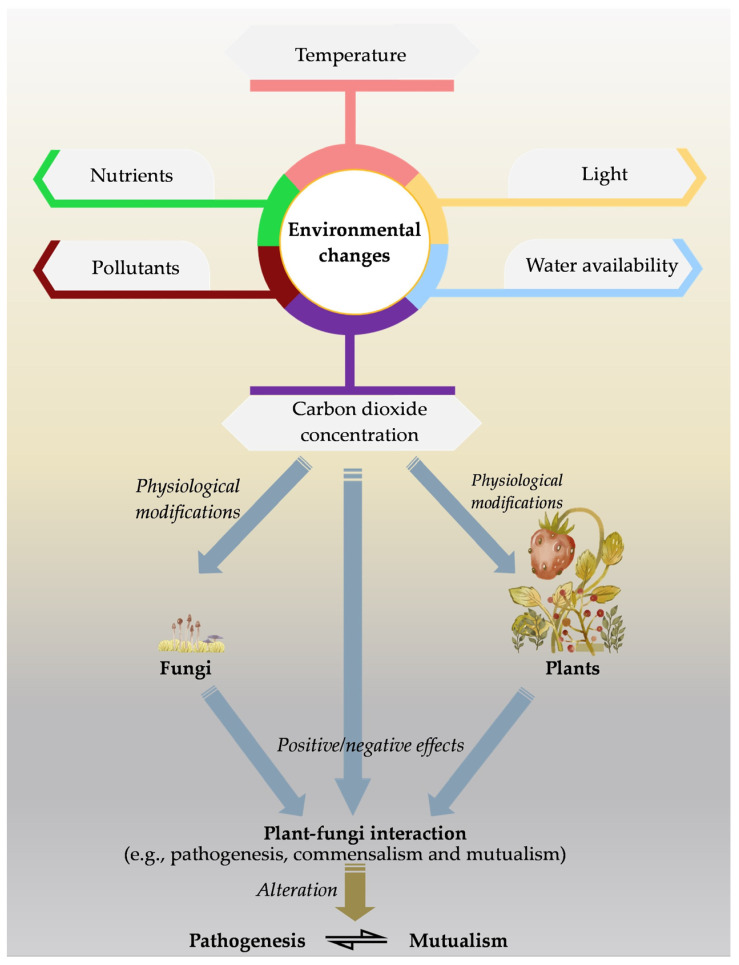
As a result of the changing environmental conditions, the physiology of plants and fungi can be affected, thereby altering the interaction between them.

Under natural settings, many of the environmental conditions can change abruptly. Therefore, the response of fungi can also change, and subsequently could have significant ramifications for interaction between the plant fungi [122]. Changes in air temperature and moisture can affect fungal physiology and metabolism since they are most sensitive to these changes [123]. The majority of soil fungi are affected directly due to those environmental changes, although changes in plant physiology, morphology, immunological response, phenological traits, and root exudation impact the fungi indirectly (Figure 1) [124]. In other words, there is a direct impact of such conditions on the plant disease resistance mechanism as well as the virulence of the pathogens [125,126]. Beneficial microbes interact with plants, need to be functional under such unbefitting conditions and improve the plant tolerance to certain stress. However, there are possibilities for shifting those mutualistic interactions to non-convenient status [127,128]. There is also a possibility for the emergence of new pathogens as altering their virulence system, potentially leading to the downfall of *R* gene-mediated plant resistance. Such emergence has also been reported, e.g., *Puccinia striiformis* f.sp. *tritici* and *Fusarium graminearum* [124,129].

#### 4.1.1. Temperature 

Rising temperatures trigger a series of cellular processes and the production of heat shock proteins, which reduce plant cell damage. In contrast, heat stress worsens cell machinery and alters chromatin modifications in plants. It also increases membrane fluidity, which causes a reaction series to become uncoupled and disrupt metabolism, thus becoming more vulnerable to the pathogens [130]. On the other hand, there is a clear surfeit of consensus among researchers that higher and lower temperatures obviously affect fungal colonization and hyphal length. However, in most cases, the elevated temperatures would be beneficial to the fungi, particularly to mycorrhizal fungi. According to the literature, such temperature change has little positive effect towards the EcM fungi, but a strong positive effect on the AM fungi. One possible reason for this could be the faster plant C allocation to the rhizosphere where these fungi live [131,132]. 

More precisely, however, each plant–fungal interaction has a unique optimum temperature range. In this regard, a number of studies have assessed the mutualistic symbiosis between plants and the mycorrhizal fungal—phylum Glomeromycota. Of this, it was found that the fungal group is more prone to cold temperatures, and instead, a warming climate enhances its growth. For instance, Mathur et al. [133] observed that a mixture of *Glomus* species (e.g., *Rhizophagus irregularis*, *Funneliformis mosseae*) colonized about 75–80% in maize under the ambient condition, but reduced to 40–45% with higher temperature, which is about 43 °C. A comprehensive study conducted by Martin and Stutz [134] found that colonization of *Glomus intraradices* in pepper (*Capsicum annuum*) roots is higher in the 20.7–25.4 °C temperature range, while colonization is minimal with temperature ranges; 32.1–38 °C. In a supportive study, Liu and colleagues [135] revealed that lowering the temperature would lead to the failure of the colonization of *G*. *intraradices* wherein 15 °C would significantly reduce the mycelial development and further completely inhibit at 10 °C. In another study, de Vallavieille-Pope et al. [136] showed the highest infection ability of wheat pathogen *Puccinia striiformis* at 10 and 15 °C. However, inactivity occurred under the warmest (20 °C) and coldest (5 °C) temperatures. According to published research findings, even if the optimum temperature condition (thus reduced colonization) is not reached, the available fungi can give considerable benefits to the plants and allow them to tolerate critical environmental conditions [137,138,139]. 

Mathur et al. [133] experimented about the effect of high temperature (43 ± 0.2 °C) on maize and also the protective role of AM fungi, *Rhizophagus irregularis*, *Funneliformis mosseae*, and other *Glomus* species. This study’s findings revealed that interactions with mycorrhizal fungi helped the plants to tolerate high temperatures while maintaining the stability of their photosynthetic apparatus—photosystem (PS) II and PSI. Furthermore, results showed that under normal conditions, mycorrhizal colonized plants had higher total chlorophyll (46 ± 1 SPAD Units) content than control plants (41 ± 1 SPAD Units). However, more elevated temperature stress plants and ultimately drop the chlorophyll contents (20 ± 1 SPAD Units), though mycorrhizal association helps the plant to recover from such conditions (37 ± 2 SPAD Units). In contrast, it is also worthwhile to mention that the heat stress turns endophytic *Botryosphaeria dothidea* into the pathogenic form, causing sudden disease severity conditions. In particular, this is common in Botryosphaeriaceae where the species of the family are recognized as stress-associated pathogens [140]. 

#### 4.1.2. Light

Ballhorn et al. [141] have inoculated the group microorganisms (*Glomus aggregatum*, *G. clarum*, *G. deserticola*, *G. etunicatum*, *G. monosporus*, *G. mosseae*, and *Gigaspora margarita*, *Paraglomus brasilianum*, *Rhizophagus irregularis*) with Lima bean (*Phaseolus lunatus*) in order to assess the impact of them under the full light and 50% shading conditions. After a 14-week trial, the researchers discovered that below-ground symbionts had boosting effects on growth and reproduction under full light. In contrast, infected plants under shaded conditions saw decreased plant growth and reproduction. This reduced plant growth is probably caused by the high C cost of the symbiosis in comparison to the available C and the inability of plants to properly offset the fungal C demand in low light [122]. On the other hand, as mycorrhizal plants decline rapidly with decreasing light intensity, P uptake by roots becomes marginal, thus leading to poor growth and development of plants [122,142]. In a recent study, Garnica et al. [143] demonstrated the impact of light on the development of root endophyte *Serendipita herbamans* in knotweed (*Reynoutria* spp.). The researchers have noticed that approximately 20% light levels increase the colonization ability of pathogens, which is also significant; however, it resulted in lower biomass (decreased by 10%) of plants compared to the control treatment. Furthermore, results show that endophyte inoculation decreased chlorophyll content by 5% (*p* = 0.020) under this condition.

In an interesting study, Hevia et al. [144] recognized the microbial biological clock-mediated plant-pathogenic fungi interaction for the first time. The researchers have used *Botrytis cinerea*, which infected *Arabidopsis thaliana* and disrupted the circadian oscillator of the fungi by providing light and dark cycling conditions. The fungal clock is the major factor influencing the outcome of the interaction between the *Arabidopsis* and *Botrytis* species. The researchers reported that the fungus may grow to its most virulent state even at daybreak, provided that its internal clock reads dusk time, thus fungal virulence potential can bypass a plant’s natural defense mechanisms.

#### 4.1.3. Water Availability

Too little water (underwater deficit or osmotic stress) or too much water (during flooding) can greatly affect many aspects of plant and microbe biology. In general, lack of water (drought conditions) restricts mycelial growth and limits its ability to supply nutrients to plants. On the contrary, plants suppressed the symbiont fungi by limiting C flow to the roots, increasing saprotrophic fungi and slightly affecting the pathogenic fungi [145]. However, such a scenario depends on the type of host plant and the associated fungi, type of soil and the stage of the plant life cycle (e.g., seedling, young, matured) [146,147]. Augé [148] showed that under drought conditions, mycorrhizal fungi could give extra tolerance to plants, also because of the higher water uptake ability. Morte et al. [149] found that colonization of mycorrhizal fungi (associated with Aleppo pine—*Pinus halepensis*) was not affected by the lowering of the feasible water content. Moreover, they have recognized that mycorrhizal interactions help Aleppo pine to overcome water stress. In contrast, Boczoń et al. [150] further highlighted that the water shortage can shift the endophytic phase of *Cenangium ferruginosum* to phytopathogenic and saprotrophic lifestyles. This stressed environment activates *C. ferruginosum* and causes pine dieback disease as the plant reduces its resistance mechanism against the pathogen. Nevertheless, it is enthralling to know how mycorrhizal fungi take up the necessary water. According to Boczoń et al. [150] this could have happened because of the ability of mycorrhizal hyphae to explore small water pores in the soil, where plant roots are not accessible, thus improving plant water status under low water availability. Added to this explanation, Bennett and Classen [132] reported that mycorrhiza support the plant via improved apoplastic water flow facilitated water uptake through fungal water channels, increased stomatal conductance in host plants, and modified host gene expression of drought-related genes encoding plant aquaporins. Moreover, it has been recognized that symbiotic endophytic fungi help plants to survive this hostile condition by increasing sugar content in cells, which enhances the osmotic adjustment by limiting drought-induced damage to the host plant. In addition, it mitigates the buildup of drought stimulated destructive H_2_O_2_ and saves plants from cellular damage [151].

In a separate study, Andreo-Jimenez et al. [152] found that under normal environmental conditions, instead of members of the Glomeromycota forming mycorrhizal associations, fungi belonging to the Zygomycota, Ascomycota and Basiodiomycota were present in rice (*Oryza sativa*). However, along with drought, the composition of the endophytic fungal microbiota changes and increases the proportion of the Ascomycota and Basidiomycota. Moreover, Andreo-Jimenez et al. [152] examined the effect of the Ascomycota fungus—*Arthrinium phaeospermum* on rice growth and found a significant correlation between higher plant yield under drought conditions. Furthermore, the early work of Stenström [153] opened a new window as he tested the impact of a water-logged environment on the fungal associations of five species with *Pinus sylvestris* seedlings. He found that *Suillus bovinus* and *S. flavidus* are highly sensitive to flooding and the reverse was true in *Hebeloma crustuliniforme*, *Laccaria laccata*, and *Thelephora terrestris*. 

#### 4.1.4. CO_2_ Concentration

The rise of CO_2_ is a universal concern. The effect on fungi is uncertain since they are both for and against depending on the group of the microorganism—among them, symbionts are generally promoted by such conditions [154,155]. In the natural environment, the climbing of CO_2_ (up to a certain level) enhances the photosynthesis of plants and improves C assimilation and allocation to roots. The root-associated microbiota favors this situation—AM fungi as they receive a higher amount of photosynthates before other soil microbes, thus higher the proliferation [156]. Nonetheless, in vitro assay conducted by Baazeem et al. [157] observed that an elevated CO_2_ (also with little raised water supply) level speeds the colony growth of *Aspergillus flavus* as well as the metabolite activities. According to their findings, when exposed to CO_2_ at 1000 ppm (37 °C), the fungi produced a substantial amount of the secondary metabolite—aflatoxin B1. This made it clear that the microbe’s metabolic activity had changed due to this unfamiliar condition, therefore the changing interaction between plants and fungi is obvious. A Meta-analysis conducted by Dong et al. [155] found increased mycorrhizal plant biomass (+26.20%), nutrient contents (N: +2.45%, P: 10.66%), and mycorrhizal fungal growth (extraradical hyphal length: +22.87%, mycorrhizal fungal biomass: +21.77%) due to the elevated CO_2_.

In a field experiment, Garcia et al. [158] attempted to understand the mycorrhizal dynamics under increased CO_2_ (200 ppm) in a warm temperate (above 4 °C) condition. In more detail, they have shown the EcM root colonization increased significantly (by 14%) under increased CO_2_, whereas the length of the AM fungi hyphae and the stocks of its glomalin (an obstinate glycoprotein that persists in the soil even after the fungus has died) concentration did not significantly change in response to CO_2_ enrichment, and the effects of CO_2_ on AM fungi root colonization varied by date. For instance, compared to the ambient CO_2_ treatment, glomalin concentrations in the elevated CO_2_ treatment tended to be 6% higher in one month, while 29% lower in another. It is quite interesting to bring the possible effect of those to plants. In this regard, Matamala and Schlesinger [159] assessed the biomass of fine roots of pine (*Pinus taeda*) forests and found biomass enhancement by 87% under elevated CO_2_. More precisely, Brosi et al. [147] evaluated the effect of elevated CO_2_ conditions on the symbiotic association between tall fescue (*Lolium arundinaceum*) and endophytic fungi *Neotyphodium coenophialum*. The researchers found that endophyte infection frequency changed under the elevated CO_2_ condition, 81% (±3) during the ambient and 91% (± 2) increased CO_2_ level, thus promoting this grass–fungal symbiosis.

Contrarily, Váry et al. [160] conducted a study to evaluate wheat Fusarium head blight (FHB) and Septoria tritici blotch (STB) disease under the elevated CO_2_. The authors reported an increased level of pathogenicity of both the *Zymoseptoria tritici* and *Fusarium graminearum* elevated CO_2_ (780 ppmv) condition compared to the optimum CO_2_ conditions (390 ppmv). Further, pathogen and plant acclimation to elevated CO_2_ leads to the rapid development of STB and FHB disease and its severity on the plant. It is also recognized that the overall pathogen acclimation to elevated CO_2_ had a greater effect on FHB development than on STB disease.

#### 4.1.5. Pollutants

Changes in environmental health via pollutants become another threat to any sort of plant–fungal interaction, where many studies have been conducted to understand the effect of heavy metals. For instance, El-Shafey et al. [161] attempted to find out the response of Rose-scented geranium (*Pelargonium graveolens*) to Cadmium (Cd)-stress. Here, further, they evaluated the effect of three endophytic fungi namely *Talaromyces versatilis*, *Emericella nidulans*, and *Aspergillus niger* on geranium and also the potentiality of the endophytes to alleviate in the changing environment. According to them, *T. versatilis* and *A. niger* had the most stimulating effects on fresh biomass of geranium leaves under normal conditions, while *E. nidulans* had the reverse impact. In this case, whereas *A. niger* only caused a non-significant increase of 14% in biomass, *T. versatilis* considerably increased biomass by 73% above the control. The biomass of non-inoculated geranium under Cd stress, however, significantly decreased to 58.4% of control. Because they considerably increased the biomass of geranium leaves to 312% and 182%, respectively, in comparison to the non-inoculated one during Cd-stress, *T. versatilis* and *A. niger*’s stimulatory impact was more evident under stressful circumstances. *E. nidulans*, on the other hand, inhibited leaf growth. Furthermore, El-Shafey et al. [161] highlighted the significant beneficial effect of the above fungi on alleviating the Cd-toxic effect of the plant by improving the resistance and enhancing the tissue quality.

Under this stressed environment, chiefly *T. versatilis* and *A. niger* stimulate the plant antioxidant enzymes, also upregulate the detoxification mechanisms of glutathione-S-transferase, phytochelatin, and metallothionein levels. In another study, Selim et al. [162] stressed the rye (*Secale cereale*) and sorghum (*Sorghum bicolor*) plants with Vanadium (V), and checked AM fungi (*Rhizophagus irregularis*) responses. Convincing data showed that interaction with *R*. *irregularis* places a beneficial effect by restricting the V intake. Further results by Selim et al. [162] noticed considerable changes in plants’ minerals content in the roots and shoots compared to *R*. *irregularis* with and without V. Accumulation of V in shoots was reduced by mycorrhizal treatment to 29% and 58% in rye and sorghum, respectively. Roots of rye and sorghum show a similar, though the more pronounced reduction in vanadium accumulation (40% and 68% reduction, respectively). Moreover, in agreement with El-Shafey et al.’s [161] findings, Selim et al. [162] reported the mycorrhizal ability to restore the plant biomass that was reduced due to the V stress. For instance, the fresh weight of the shoots and roots of the sorghum plant is noticeably increased by 216% and 158%, respectively. In contrast, the researcher also noticed the growth of the shoots and roots in rye are insensitive to mycorrhizal availability. 

#### 4.1.6. Nutrients

Deprivation of available soil nutrients also leads to a stressful environment for the plants; the priceless association with fungi may play a role in mitigating such effects on plants. In a study, Garnica and co-workers [143] recognized that under low-nutrient conditions, *Serendipita herbamans* strongly colonized knotweed and had positive effects on plant growth, where biomass increased by 15% and chlorophyll content increased by 13% (*p* = 0.006). Mycorrhizae’s role in promoting plant growth has been well-documented, markedly in conjunction with the increased uptake of P [163]. Patricia Guadarrama et al. [164] studied the effect of increasing P nutrition and mycorrhizal growth response of *Lotus corniculatus* and *L. glaber* in soil with little accessible P. Interestingly, the study revealed that there is no significant effect with the addition of P in plant shoot yield, as both mycorrhizal and non-mycorrhizal plants responded strongly to added P in soil. This shows that the interaction between fungi–plant may not be an advantage or disadvantage as a means of increased P availability. Similarly, Garcia et al. [158] noticed that N fertilization had no effect on the colonization of EcM roots though increased the colonization of AM fungi. More recent work by Garces et al. [165] found the influence of N enrichment on *Epichloë* colonization of the dune-building grass, *Ammophila breviligulata*. It is reported that the presence of *Epichloë* spp. in the host grass increases the species richness of root endophytes by 17%. The addition of N, however, exhibited no noticeable main or interaction influence on the richness of root endophytes. In contrast, N addition impacted the composition of the root endophyte population, primarily in areas where *Epichloë* spp. were prevalent.

### 4.2. Environmental Factors and Lifestyle Switching of Fungi

Most of the fungi (possibly all) can express different lifestyles, e.g., *Colletotrichum magna* is pathogenic in a variety of cucurbits, a saprophyte in dead plant parts, and a nonpathogenic endophyte in tomatoes. It is difficult to pinpoint the exact reason for this, but environmental changes could be one of the driving factors behind it [166]. The environmental factors on lifestyle switching are generally coupled and discussed with the symbiotic fungi, which often live with plants by creating mutualistic relationships; however, they may change their lifestyle into a parasitic one [167,168]. In a study, Delaye et al. [169] analyzed the lifestyle switching of endophytes, necrotrophs, and biotrophs. Accordingly, lifestyle switching of fungi is generally noticed as the conversion of endophyte to necrotroph and vice versa. Such changes are identified to happen at an equal frequency, however, endophytic to a pathogenic biotrophic lifestyle is rare. Further, once biotrophy has evolved, it is suggested that lifestyle change to another form is impossible to occur. There are clear findings of changing symbiotic endophytes into pathogens under various unfamiliar environmental conditions [170]. This can be explained as endophytes interacting with the plants via a balanced antagonism, in order to recognize the host and colonization they need to switch to the virulence mechanism, and as a result, it triggers the plant defense mechanism. Endophytes maintain equilibrium in plant interaction, while fungi survive with the nutrient exchange. However, as aforementioned, the hazardous nature of the environment affects the health of the plant, thus weakening the defense response. This makes an opportunity for the endophytes (not the true endophytes) to grow abruptly and change their life into a pathogenic form [171,172]. For instance, *Alternaria* species can become pathogenic when the plant becomes stressed and weakened [173].

Changes in the nutrient content are the most recognized environmental factor, which stimulates lifestyle transitions. Unlike nutrient deprivation, the higher nutrient condition is highly responsible for this [167]. Enriched nutrient conditions (e.g., heavy application of fertilizers in agro farming) generally improve plant growth and development. At the same time, lifestyle switching of mycorrhizal into pathogenic is noticeable as balanced mutualistic interaction becomes less balanced. Anyhow, it is difficult to conclude which nutrient (e.g., P or N) could lead to this condition, as various results appeared throughout the literature [174]. Other than the nutritional costs and benefits, Mandyam and Jumpponen [175] illustrated that *Periconia macrospinosa*, a dark septate endophyte, changes its lifestyle symbiosis to pathogenic with the increasing lower light condition/shade. In support of this, Álvarez-Loayza et al. [176] depicted that *Diplodia mutila* favors low light and endosymbiotic continues with young palm seedlings (*Iriartea deltoidea*), though high light triggers pathogenicity of the fungus owing to the enhanced produces of Melanin (correlate with increased production of ROS) and H_2_O_2_. Álvarez-Loayza et al. [176] suggested that higher light intensity increases fungus virulence by triggering the HR response in plants.

The reverse is also possible, as pathogens switch into symbiosis [167]. Of this, if the lifestyle characterization (molecular level) of the fungi is based on the capability to cause host disease resistance, stress tolerance (e.g., drought), or growth enhancement, then the parasitic lifestyle can convert into mutualistic when the right time comes. Such condition is recognized with the *Colletotrichum* species [177].

Additionally, the precise mechanism that causes this scenario to arise is yet to be resolved. However, several fragmented attempts made little understanding. For example, Hill et al. [178] worked with the lifestyle changes of *Fusarium* spp. and found that the copy number variation of gene *CSEP* and *CAZyme* is the main driving force as no significant difference in *CSEP*, *CAZyme*, or gene repertoires between phytopathogenic and endophytic strains were noticed. In a separate study, Muszewska et al. [179] highlighted that serine proteases play a crucial role in changing lifestyles.

## 5. Conclusions

Interactions between plants and fungi have become unavoidable and rather indispensable. Understanding plant–fungi interactions is essential in the current world, particularly for sustainable agriculture and food security and ultimately the well-being of humans and livestock. Plants and fungi have a complex and dynamic relationship that can be beneficial or detrimental for both partners. On one hand, some fungi are pathogenic to plants, causing diseases that reduce plant reproduction, growth, development, and productivity, ultimately affecting their persistence. On the other hand, others form symbiotic associations with plants, such as AM and endophytic fungi, which enhance plant nutrient uptake, growth, and stress tolerance. Moreover, the outcome of plant–fungi interactions can be influenced by various environmental factors, including temperature, light, water, CO_2_, pollutants, and nutrient concentration in the soil, which can modulate the balance between mutualism and antagonism. The intricate molecular mechanisms that trigger the cascade of interactions between plants and their associated fungi under environmental changes, however, are still little understood. There are only a handful of papers documenting the specific non-molecular based mechanism employed by fungi in modifying the plant physiology and themselves to protect against the changing unwelcome environmental conditions. Overall, plant–fungi interactions are a double-edged sword for plant health and survival, depending on the type of fungi involved, the physiological state of the plant, and the abiotic conditions of the habitat.

## Data Availability

Not applicable.
