# Correction: Priyashantha et al. Plant–Fungi Interactions: Where It Goes? *Biology* 2023, *12*, 809

**DOI:** 10.3390/biology13070490

**Published:** 2024-07-01

**Authors:** A. K. Hasith Priyashantha, Dong-Qin Dai, Darbhe J. Bhat, Steven L. Stephenson, Itthayakorn Promputtha, Prashant Kaushik, Saowaluck Tibpromma, Samantha C. Karunarathna

**Affiliations:** 1 Center for Yunnan Plateau Biological Resources Protection and Utilization, College of Biological Resource and Food Engineering, Qujing Normal University, Qujing 655011, China; priyashanthahasith@gmail.com (A.K.H.P.); cicidaidongqin@gmail.com (D.-Q.D.); 2 Department of Botany and Microbiology, College of Science, King Saud University, P.O. Box 2455, Riyadh 11451, Saudi Arabia; bhatdj@gmail.com; 3 Biology Division, Vishnugupta Vishwavidyapeetam, Gokarna 581326, India; 4 Department of Biological Sciences, University of Arkansas, Fayetteville, AR 72701, USA; slsteph@uark.edu; 5 Department of Biology, Faculty of Science, Chiang Mai University, Chiang Mai 50200, Thailand; itthayakorn.p@cmu.ac.th; 6 Independent Researcher, 46022 Valencia, Spain; prakau@alumni.upv.es; 7 National Institute of Fundamental Studies (NIFS), Hantana Road, Kandy 20000, Sri Lanka

Affiliation Update:

In the original publication [1], there was an error regarding the affiliation for Prashant Kaushik. The updated affiliation should be: Independent Researcher, 46022 Valencia, Spain.

The authors state that the scientific conclusions are unaffected. This correction was approved by the Academic Editor. The original publication has also been updated.
